# Fowl Adenovirus Serotype 1: From Gizzard Erosion to Comprehensive Insights into Genome Organization, Epidemiology, Pathogenesis, Diagnosis, and Prevention

**DOI:** 10.3390/vetsci12040378

**Published:** 2025-04-17

**Authors:** Amina Kardoudi, Abdelouhab Benani, Abdelmounaaim Allaoui, Faouzi Kichou, Latefa Biskri, Ikram Ouchhour, Siham Fellahi

**Affiliations:** 1 Department of Veterinary Pathology and Public Health, Agronomy and Veterinary Institute Hassan II, Rabat-Instituts, Rabat 10101, Morocco; f.kichou@iav.ac.ma (F.K.); ouchhourikram@iav.ac.ma (I.O.); 2 Medical Biology Department, Molecular Biology Laboratory, Pasteur Institute of Morocco, Casablanca 20360, Morocco; abdelouaheb.benani@pasteur.ma; 3 Microbiology Laboratory/African Genome Center, Mohammed VI Polytechnic University, Ben Guerir 43150, Morocco; allaoui@outlook.be (A.A.);

**Keywords:** Fowl Adenovirus Serotype 1, FAdV-1, AGE, genome, epidemiology, diagnosis, prevention

## Abstract

The concerns regarding Fowl Adenoviruses have gained significance in the poultry industry due to their association with various diseases, including Inclusion Body Hepatitis, Hepatitis-Hydropericardium Syndrome, and Adenoviral Gizzard Erosion (AGE). AGE is an emerging disease reported in several countries, particularly in Asia and Europe, causing significant economic losses in the poultry industry. In 2001, Fowl Adenovirus Serotype 1 was identified as the etiological agent of AGE in Japan. Since then, it has been spreading to other countries due to its transmission mode. Although Adenoviral Gizzard Erosion has been mostly described in broilers, it has also been observed in layers and pullets. This review offers a comprehensive analysis of Fowl Adenovirus Serotype 1, encompassing various key aspects of the virus. We also examine the pathogenesis and epidemiology of the virus, providing an overview of its distribution and prevalence in avian populations worldwide. Highlighting the most recent developments in serological and molecular techniques for virus detection, quantification, and genotyping and comparing them to conventional tests, this review aims to contribute to the understanding of the diagnostic workflow for this virus. Lastly, this review sheds light on some vaccine strategies to prevent Adenoviral Gizzard Erosion.

## Simple Summary

Adenoviral Gizzard Erosion (AGE) is a disease affecting poultry, causing significant economic losses, especially in broiler chickens, and is caused by Fowl Adenovirus Serotype 1. AGE does not present typical symptoms but can be associated with general signs such as weight loss, depression, reduced growth, and sometimes higher mortality. This review also presents the diagnostic workflow for FAdV and highlights the advantages of molecular techniques over conventional methods. Molecular techniques offer higher specificity, faster results, and greater sensitivity, enabling accurate detection of the virus. The review also covers vaccination strategies to control the spread of AGE. The findings from this review will assist farmers in developing more effective prevention and vaccine strategies, ultimately improving poultry health and productivity.

## 1. Introduction

Among the twelve serotypes of Fowl Adenovirus (FAdV), Fowl Adenovirus Serotype 1 (FAdV-1) occupies a noticeable position as it has been confirmed as the etiologic agent of Adenoviral Gizzard Erosion (AGE) following the epizootic outbreak in broiler flocks reported in western Japan in 1989 and 1999 [1]. AGE is a serious disease typically characterized by erosions and ulcerations of the gizzard mucosa, reducing flock performance [2]. Understanding the structural and molecular characteristics of FAdV-1, as well as its physicochemical properties and modes of transmission, is imperative for implementing effective control strategies and developing preventive measures to protect the health and productivity of farms. This review emphasizes the critical role of FAdV-1 as the etiological agent of AGE and provides an in-depth overview of the virus’s biology, genomic organization, epidemiology, and worldwide distribution, along with insights into its pathogenesis. We also examine recent advancements in molecular and serological techniques for FAdV-1 diagnosis and compare them to classical diagnostic methods. Finally, the review delves into vaccine strategies to prevent AGE. By shedding light on the multifaceted aspects of FAdV-1, we contribute to a better understanding of the virus and its associated disease, guiding efforts to manage AGE effectively within the poultry industry.

## 2. Classification, Structure, and Physicochemical Properties of FAdV-1

Fowl Adenoviruses are non-enveloped viruses that are medium-sized, typically measuring 70–90 nm in diameter. They belong to the *Aviadenovirus* genus within the *Adenoviridae* family [3,4]. The *Aviadenovirus* genus is categorized into five species—Fowl Adenovirus A to Fowl Aadenovirus E (*F*AdV-A to -E)—based on their distinctive profiles of restriction enzyme digestion [5], and twelve serotypes (FAdV-1-8a to 8b-11) using Viral Neutralization (VN) [6]. Several impactful poultry diseases, including AGE, Hepatitis-Hydropericardium Syndrome (HHS), and Inclusion Body Hepatitis (IBH), are associated with specific FAdV serotypes. AGE is caused by FAdV-1 [1,7,8], HHS is caused by Fowl Adenovirus Serotype 4 from species C [9,10], and FAdVs associated with IBH belongs to species D and E [11,12]. FAdV-1 is one of twelve FAdV serotypes grouped within species A, which is monoserotype. The FAdV-1 particle has a buoyant density of 1.35 g cm^3^ in cesium chloride (CsCl) and is composed of around 17.3% DNA and 80.7% protein [13]. Like other FAdVs, FAdV-1 is composed of ten major structural proteins and eleven non-structural ones [8,14]. These proteins are surrounded by hexameric and pentameric capsomeres arranged in a pattern of icosahedral symmetry [15]. The hexameric capsomer forms the icosahedral capsid’s facets, while the pentameric capsomere caps the vertices of the capsid. The latter comprises two different types of proteins: a penton base anchored in the capsid, and a fiber protein forming a direct complex with the penton protein. Two distinct-length proteins have been identified exclusively in FAdV-1, FAdV-4, and FAdV-10, which are attached to a single penton base [16,17,18]. According to [16], the long fiber (Fiber 1) of Chicken Embryo Lethal Orphan (CELO) is composed of 793 amino acids (aa), while the short fiber (Fiber 2) consists of 410 aa. In contrast, Fiber 1 of FAdV-4 consists of 431 aa, while Fiber 2 is composed of 479 aa [19].

The virus is remarkably stable in storage and is insensitive to slightly acidic conditions, as well as to lipid solvents [13]. As a result, the virus resists a variety of common disinfectants and remains infectious in the environment for a long period of time. This suggests that the type of disinfectant and the techniques employed for building and equipment disinfection are crucial, particularly when AGE outbreaks reappear on specific farms.

## 3. Genome Organization of FAdV-1

FAdVs have the largest genomes among members of *Adenoviridae* family [4]. This property, plus the low pathogenicity of some FAdV serotypes, makes them attractive as recombinant vaccines or gene transfer vectors [20]. Overall, FAdV-1’s genome is very similar to that of other FAdV serotypes, showing a linear genome with a double-stranded DNA molecule of 43.8 kb, which is around 8 kb longer than the genomes of Human Adenoviruses of subgenus C Ad2 and Ad5 (35.9 kb) [21]. FAdV-1’s genome size is slightly different between strains (Reference Strain/CELO (43,804 kb), Strain/W-15 (43,849 kb), Strain/JM1/1 (43,809 kb), Strain/SVA16ALD002867 (43,856 kb)) [16] (Table 1). In addition, FAdV-1’s strains are known for their genetic stability and share over 99% nucleotide similarity [22]. Genetic diversity within FAdV-1 results mainly from the progressive accumulation of point mutations and indel mutations, mainly in the tandem repeats regions [23]. However, mutations resulting in amino-acid changes in the coding regions of FAdV-1 genomes are uncommon [22,24].

The genome of FAdV-1, like other FAdV serotypes, is composed of multiple distinct regions that contain both coding and non-coding elements, which are essential for its replication and genome packaging. The coding regions of the genome include various open reading frames (ORFs), each with specific functions. These ORFs are organized from 5′ to 3′ as follows: ORF 0, ORF 1 (dUTPase homologous), ORF 1A, ORF 1B, ORF 1C, ORF 2, ORF 14, ORF 13, ORF 12, IVa2, pol, pTP, 52K, pIIIaIII (penton base), pVII, PX, pVI, Hexon, Protease, DBP, 100K, 33K, 22K, pVIII, Hexon, Fiber-1, Fiber-2, ORF22, ORF20A, ORF20, ORF8, ORF17, ORF16, ORF9, ORF10, ORF11, and ORF26 [25].

The central part of the FAdV-1 genome, spanning from the IVa2 gene (at 5000 nt) to the fiber genes (at 33,000 nt), follows the same organization as Mastadenoviruses [14,16]. It encodes structural proteins such Hexon, Penton base, IIIa, Fiber1, Fiber 2, pVI, pVII, pVIII, and pTP as well as non-structural proteins including 100 kDa, 52 kDa proteins, and DNA Polymerase. Studies have reported that the FAdV-1 DNA Polymerase gene offers an alternative to the hexon gene, providing an interesting option for assessing phylogenetic variability and epidemiological relationships between FAdV-1 strains [26]. Surprisingly, the CELO strain genome has 5 kb at the left end and 15 kb at the right end, with no sequence similarity to the Ad2 early regions E1, E3, and E4, or the PV and PIX proteins [16]. This could be due to a possible rearrangement or inversion of the genome ends, making the classic E1, E3, and E4 regions undetectable [3].

Like other adenoviruses, the FAdV-1 genome contains several non-coding regions, including the Inverted Terminal Repeats (ITRs) and the packaging sequence [16]. These regions are vital for the virus’s replication and genome packaging processes. The ITRs, located at the extremities of the genome, play a crucial role in ensuring proper replication and packaging of the viral genome. Interestingly, the ITRs of the CELO virus are relatively short compared to those of the human adenovirus HAdV-5 (54 bp vs. 103 bp, respectively) [27].

The unconventional presence of G nucleotides at positions 1, 4, and 7 of the ITRs of some FAdV-1 isolates has been reported [28]. Strikingly, while most Aviadenovirus ITR sequences begin with 5′-CATCATC, an exception exists in avian adenovirus 1 (FAdV-1). The PHELPS isolates (GenBank accession no. U46933) [16] and KUR (GenBank accession no. M57604) are unique among Adenoviruses whose genomes begin with the 5′-GATGATG sequence. However, other FAdV-1 isolates, OTE (GenBank accession: K00939 and K00940), 61/11z (GenBank accession: KX247012), and W-15 (GenBank accession: KX247011), are distinct from PHELPS and KUR as they conform to the Ads convention and begin with the sequence 5′-CATCATC [2].

## 4. Epidemiology of Adenoviral Gizzard Erosion

The first description of AGE associated with necrotizing pancreatitis was reported by [29]. This study was conducted on a natural outbreak affecting pullets aged 60 to 70 days, using immunohistochemistry and Electron Microscopy (EM) [29]. Subsequently, adenoviral lesions of the gizzard were observed in other studies involving pullets and broiler flocks in the United States [30], as well as in an outbreak with increased mortality among Japanese broilers [31], without specifying the responsible agent. However, following an outbreak of AGE in broiler chickens reported in western Japan between 1989 and 1999, FAdV-1 was characterized by PCR/RFLP for the first time as the etiological agent of AGE [1]. Although sporadic isolates of FAdV-8a, -8b, or -4 were isolated from affected gizzards [32], the majority of subsequent AGE outbreaks could be attributed to FAdV-1 [8]. The disease is not limited to Japan as it has been reported in other countries, particularly in Asian countries, including Korea [33], China [34], Malysia [26], Iran [35], Brunei [26], and India [36]. Moreover, the disease has also been detected in European countries, such as Italy [37], Germany [38], Denmark [26], Greece [26], Sweden [8], Poland [2], Hungary [39], Belgium [40], Slovakia [41], and Great Britain [7], as well as other countries, like Egypt [38] (Table 1).

On the other hand, FAdV-1 has been isolated from a collection of healthy chicken samples from different countries including Indonesia, Ukraine, Canada, Morocco, Peru, USA, Saudi Arabia, South Africa, Mexico, and Romania [26]. These isolates were not associated with AGE, as the chickens showed no clinical symptoms or gizzard lesions. The rapid spread of FAdV-1 is associated with its mode of transmission. FAdV-1 can be transmitted both vertically and horizontally. Horizontal transmission of the disease was documented in an experimental study conducted on 13-day-old SPF chickens, through direct contact with chickens inoculated with a pathogenic strain (99ZH) [42]. This type of transmission is related to the stability of the virus in the environment and its tolerance to cold, acids, leavening agents, and UV light. In addition, tolerance to the global changes in rearing systems, such as replacing traditional cage rearing systems with a barn system, also play an important role in the field epidemiology as the pathogen could be more easily distributed between flocks.

In addition, vertical transmission has been highlighted as an essential pathway of FAdV-1 propagation [43]. In the early 1950s, the CELO virus was isolated from chicken embryos, which indicates that it is an endogenous virus transferred from the parent bird to the embryonated egg [44]. Subsequently, FAdV-1 was isolated from uninoculated fertile eggs and the virus reappeared in cell cultures prepared from vertically infected embryos [45]. This underlines the importance of the vertical transmission of FAdV-1. Parent birds can serve as a reservoir for the virus, from which FAdV-1 may be introduced into descendant flocks via transmission from embryonated eggs. In Germany, for the first time, FAdV-1 was directly detected in gizzard embryos and offspring from a seropositive broiler breeder, even in the presence of high levels of neutralizing antibodies [43]. In contrast, in Sweden, the virus was not identified in 277 embryo samples from parent birds with high levels of neutralizing antibodies [8].

Geographically, AGE disease has mainly been described in broilers from Europe and Asia, causing enormous economic loss [24]. Although mortality has been observed in some broiler flocks [46], most documented AGE outbreaks have primarily resulted in problems such as growth retardation [8]. However, AGE outbreaks in laying hen flocks are accompanied by increased mortality, suggesting a significant impact on bird health and production [2] (Table 1).

**Table 1:** Description of Adenoviral Gizzard Erosion Outbreaks associated with FAdV-1 in some countries.

Country	Year	Production Type	Age	Organ	Clinical Symptoms/Pathological Findings	Co-Infection	Diagnosis	Isolated Strain Name	Genome Size	**Access Number**	**Reference**
**Japan**	1998–1999	Broiler	51 days	Gizzard mucosa	-No clinical signs. -2% to 3% mortality rate.	None	AGE	99ZH	NR	LC504224.1	[1]
	2000	Broilers	NR	NR	-Gizzard erosion.	None	AGE	JM1/1	43,809	MF168407	[23]
Korea	2010	Layer	150 days	Gizzard	-Dullness, anorexia, and emaciation. -0.2% mortality rate per week.	None	AGE	K181	NR	JN181575 ^Hg^	[33]
Hungary	2010	Layer	25 weeks	Gizzard	-Gizzard ulcer and clotted blood in esophagus, proventriculus, and gizzard.	None	AGE	D1504/2/10/HU	43,726	OP985604	[26]
	2010	Layer	22 days	Gizzard	-Clotted blood, gizzard ulcer and erosion.	None	AGE	D1552/10/HU	43,801	OP985605	[26]
	2014	Breeder	6 weeks	Proventriculus	-Gizzard ulcer, pancreas necrosis, enlarged liver.	None	AGE	D2604/2/14/HU	43,841	OP985621	[26]
	2019	Broiler	4 weeks	Gizzard	-Gizzard erosion and ulcer.	MDV-1	AGE	D4622/2/19/HU	43,840	OP985634	[26]
Brunei	2010	Layer	27 weeks	Gizzard	-Black vomit (clotted blood), gizzard erosion, severe congestion at proventriculum–gizzard junction.	None	AGE	D1499/3/10/BN	43,817	OP985603	[26]
Malaysia	2011	Broiler	35 days	Cecal tonsil	-Swollen head, enlarged liver, ascites. -8% mortality rate.	Reovirus	AGE	D1726/1/2/11/MY	43,794	OP985608	[26]
	2018	Broiler	23 days	Proventriculus	-Flabby proventriculus, gizzard erosion, high mortality.	None	AGE	D4182/18/MY	43,882	OP985630	[26]
China	2019	NR	NR	Gizzard	-No clinical signs. -No mortality.	None	AGE	CH/HNWC/1905	NR	MZ435837	[34]
Poland	2011	Laying hens	38 weeks	Gizzard	-Decreased egg weight and production. -2.3% mortality rate.	None	AGE	Wroclaw-15 (W-15).	43,849	KX247011	[2]
	2008	Broilers	NR	Proventriculus, gizzards, and intestines	-Uneven growth, depression, and dull feathers. -No mortality.	None	AGE	PL/068/08	NR	GU952109 ^Hg^	[24]
	2013	Layer	25 weeks	Gizzards, duodenum, and livers	-No clinical signs. -0.4% mortality per week.	None	AGE	PL/G018/13,	NR	KY027457 ^Hg^	[47]
	2013	Layer	25 weeks	Gizzards, duodenum, and livers	-No clinical signs. -0.25% mortality per week.	None	AGE	PL/G026/12	NR	KY027456	[47]
Great Britain	2009–2016	Replacement pullets and layers	41 days	Gizzard	-No clinical signs. -0.12–0.30% mortality per week.	None	AGE	NR	NR	NR	[7]
Greece	2014	Broiler	NR	Gizzard	-No specific symptoms.	None	AGE	D2563/4/14/GR	43,787	OP985620	[26]
Germany	2011	Broilers	19 weeks	Gizzard	-Uneven growth. -No mortality.	None	AGE	11/7127	43,795	MK572848	[43]
	2011	Broilers	15 to 36 days	Gastric mucosa	-Reduced daily weight gain. -Reduced feed intake, and lack of uniformity in flocks. -8% mortality.	None	AGE	G11-1588	NR	NR	[46]
India	2013–2014	Commercial layer	4 to 12 weeks	Gizzard proventriculus, liver, and pancreas	-Dullness, uneven growth, and decreased feed and water intake. -10 to 30% mortality rate.	CAV	AGE	PDRC-NZ-145-2013	NR	NR	[36]
Belgium	2014	Commercial broiler	21 days 25 days	Gizzard, liver, and pancreas.	-Depression, reduced feed intake, reduced weight gain, and lack of uniformity in flocks. -No mortality.	None	AGE	NR	NR	NR	[40]
Denmark	2016	Broiler	24 days	Gizzard	-Gizzard erosion.	None	AGE	D3678/2/16/DK	43,788	OP985628	[26]
Sweden	2016	Broiler	16–19 days	Gizzard, liver, and caecal tonsils.	-Loss of appetite, decreased growth, uneven size.	None	AGE	SVA16ALD002867	43,856	MW054563 MW054564 MW054566 MW054565 MT133691	[8]
Egypt (Beheira)	2020	Broiler	NR	Cloacal swabs	-Gastrointestinal disorders. -Loss of weight.-Depression. -No mortality.	None	AGE	AD17	NR	MW689188 ^Hg^	[38]
Slovakia	2023	Broiler	15 days	Liver and gizzard	-Depression, apathy, somnolence, crouched position with droopy head, fuzzy feathers, anemic combs and wattles, sporadic nervous signs, and reduced weight gain.	None	AGE	NR	NR	NR	[41]
Iran	2019	Broiler	16 days	Gizzard	-Depression, weight loss, and lack of flock uniformity. -6% mortality rate.	None	AGE	IRMGH019	NR	MN165288 ^Hg^	[35]
Italy	1995 to 2006	Broiler Layers	42 to 63 days	Gizzard	-No clinical signs.	None	AGE	NR	NR	U46933	[37]

Hg: Hexon gene, NR: not reported, AGE: Adenoviral Gizzard Erosion, MDV: Marek’s Disease Virus, CAV: Chicken Anemia Virus.

## 5. Clinical Manifestations and Predisposing Factors

The search for the underlying causes of AGE has evolved over time. Initially, research focused on factors such as dried green foods [48], feed structure and fiber [49], starvation [50], histamine ingestion [51], nutritional deficiencies in vitamin B6 [52], and high levels of fish meal [53]. Later, attention shifted to harmful substances such as gizzerosine [54] and mycotoxins [55], and eventually to infectious agents such as *Clostridium perfringens* [56] and FAdV-1 [1]. As shown in Table 1, AGE does not present typical clinical signs. In most cases, the disease manifests mainly as weight loss, leading to reduced flock uniformity. Occasionally, symptoms are accompanied by depression, feathering, dullness, anorexia, and decreased egg production in laying hens [40]. However, the impact on egg production remains controversial. No significant impact on egg quality or production has been reported in broiler [23] or laying flocks affected by FAdV-1 [7]. In contrast, a significant decrease in egg production, along with a reduction in egg size, has been observed during AGE outbreaks in old layers [2]. In some cases, clinical signs are accompanied by mortality rates reaching up to 8% in infected chickens [2]. This mortality has been shown to be related to deep ulcerations and perforation of the gizzard, resulting in heavy bleeding in laying hens in Poland [2]. In other cases, high mortality rates have been reported without clinical signs [7] (Table 1).

Experimental infections with strains isolated during field epidemics have been reproduced in SPF laying hens, SPF broilers, and commercial broilers [57]. Most experimental studies have reported no clinical signs or only mild clinical signs following FAdV-1 infection. However, the macroscopic and histological characteristics of AGE, such as degeneration and necrosis of glandular epithelial cells accompanied by the presence of intranuclear inclusion bodies, have been described simultaneously with the detection of FAdV DNA.

Several factors influencing the development of AGE have been examined in experimental studies. Clinical signs and pathological lesions were demonstrated to be dependent on the quantity of FAdV-1 inoculated into broilers and commercial chickens experimentally infected with a virulent European strain [58]. However, the influence of different diets designed to promote gizzard stimulation was excluded, as all SPF broilers experimentally infected shows gizzard lesions, regardless of their diet [59]. Furthermore, it has been suggested that immunosuppression by other FAdV serotypes, some pathogens such as Infectious Bursal Disease Virus (IBDV), and immunosuppressive drugs such as cyclophosphamide (CY) facilitate the development of gizzard erosion induced by FAdV-1 [60]. Chickens pre-treated with other pathogens have been shown to have significantly higher gizzard lesion scores than untreated birds. Domanska-Blicharz et al. described an atypical pathological development of the disease, where day-old SPF layers infected with FAdV-1 via the ocular and nasal routes showed mortality rates of up to 100% and severe gizzard erosions, while 21-day-old infected birds showed no clinical or pathomorphological signs of AGE [24]. This suggests that older birds develop a resistance against infection by FAdVs. However, other studies have excluded the influence of birds’ age at the time of inoculation on AGE development [61]. Finally, it has been shown that the development of AGE is influenced by the FAdV-1 strain itself. Strains isolated during an AGE outbreak, such as Tokushima2000/GE, 99-ZH, PA7127, and JM1/1, were able to induce pathological lesions characteristic of AGE in broilers and commercial chickens [61]. However, other strains, such as CELO and OTE (Section 4), did not induce AGE [61].

## 6. Mechanism of Infection

The mechanism by which FAdV-1 induces AGE remains unclear [62]. It has been documented that FAdV can bind to different molecules such as integrins (Atb3 or Atb5), CMH class I particles, proteoglycans, and other undetermined receptors in the lungs and nervous system [32]. An initial study demonstrated that FAdV-1 interacts with a protein of around 200 kDa expressed in the gizzard using Virus Overlay Protein Binding Assay (VOPBA) [63]. Subsequently, the Coxsackievirus and Adenovirus Receptor (CAR) was confirmed as a target of FAdV-1 and FAdV-4 [62]. CELO transduction, defined as the capacity of a virus to attach to a target cell, penetrate it, and regulate the expression of a transgene within that cell, was found to be approximately 100-fold higher in the presence of CAR overexpressing cells, demonstrating that CELO has a CAR-dependent transduction behavior like Ad5 [62]. Several studies have highlighted the dual role of both short and long fibers in the infectivity and pathogenicity of FAdV [64]. Recently, it has been confirmed that FAdV-1 and FAdV-4 bind to the CAR receptor via Fiber-1 [64]. This interaction occurs through the fiber-1 knob and domain 1 (D1) of CAR, as verified by confocal microscopy. The involvement of CAR domain 2 (D2) in this interaction remains to be analyzed in the case of FAdV-1 [65].

## 7. Diagnosis

### 7.1. Clinicopathological Features

As mentioned in Section 5, chickens with AGE do not present typical clinical symptoms. Most previous cases came from gizzard examination directly on the slaughter line of flocks that did not show typical clinical signs [34]. However, since clinical signs such as weight loss and uneven growth of the flock are the most frequently observed, these signs have been proposed as indicators of AGE outbreaks [43].

Macroscopically, the disease manifests as hypertrophic and dilated gizzards with moderate to severe focal erosion. The lesions are characterized by a dark discoloration indicating bleeding and detachment of the koilin layer [2]. Other studies have reported the presence of bloody fluid in the gizzard as well as in the proventricular and/or intestinal lumen [8]. However, gizzard perforation has been reported in dead birds [7]. Ylva Lindgren and her team observed, for the first time, the presence of undigested feed in the intestines of affected flock birds in which FAdV-1 was isolated [8]. This discovery raises the hypothesis that AGE may be correlated with poor food digestion due to the altered function of the gizzard, which is responsible for both food grinding and motility. This may explain the growth deficiencies observed in affected broiler flocks.

### 7.2. Histopathological Diagnosis

Histopathological examination of the gizzard is a crucial step in diagnosing FAdV-1 infection before proceeding with virus isolation or other diagnostic techniques. This examination typically involves the application of standard histological staining methods, which help to evaluate the morphological criteria and functional properties of the affected tissues. In addition, other methods are now used to enhance the performance of histopathological analyses. These include immunohistochemical methods, DNA hybridization, and In Situ Hybridization (ISH) [8].

Histopathological analysis of gizzards from infected chickens often reveals moderate to severe degeneration of the mucosal layer and the koilin layer, which is typically accompanied by erythrocyte infiltration and the presence of inflammatory cells, including granulocytes, mononuclear cells, and lymphocytes in the lamina propria, submucosa, and muscle layers [36]. The presence of intranuclear inclusion bodies, a hallmark of avian adenovirus, is commonly observed during outbreaks [8]. These inclusion bodies were seen in degenerated cells in the near-surface koilin layer of broilers in Sweden as well as in the glandular epithelium of laying and replacement chickens in Britain [8]. However, the absence of inclusion bodies with typical adenoviral morphology does not rule out the presence of FAdV-1, as they can be transitional and may sometimes be difficult to identify. In this case, to confirm the presence of FAdV-1, more sensitive techniques such as EM are highly recommended, as they enable direct visualization of viral particles in icosahedral form in allantoic fluid or affected tissues [8].

### 7.3. Virus Isolation

The isolation of FAdV is a crucial step in the investigation and diagnosis of viral infections in birds.

Although this procedure is time-consuming, it remains a benchmark for confirming FAdV infection. This step is also necessary for studying the pathogenicity of the strain in SPF chickens or embryos, as well as for sequencing and characterizing the entire genome. To achieve that, various types of cell culture have been used. In the majority of studies, FAdV-1 is isolated on Chicken Embryo Liver (CEL) cells [7]. In other cases, Chicken Embryonic Kidney (CEK) cell cultures are used [66]. Alternatively, it is possible to isolate FAdV using the Chicken Hepatoma cell line (LMH) [6]. More recently, successful isolation of FAdV-1 have been carried out using embryonated SPF chicken eggs and harvesting liver homogenates [8].

### 7.4. Serological Techniques

Several serological techniques have been employed for the diagnosis of FAdV, including FAdV-1. Initially, from prepared hyperimmune sera, Agar Gel Immunodiffusion (AGID) [67], Double Immunodiffusion (DID) [68], Counter-Immunoelectrophoresis [69], and AGP tests [70] have been used for FAdV detection in different tissues of infected birds. A microtiter fluorescent antibody test has also been developed for serological detection of FAdV infection in chicken [71]. This test has shown higher sensitivity than the DID test. Alternatively, an Immunocytochemical Assay using the Avidin-Biotin-Peroxidase complex has been shown to be an efficient way of detecting FAdV antigens in a variety of tissues [72]. Although these tests are cheaper, rapid, and more adapted to mass detection, they present several limitations in terms of cross-reactivity with other virus groups, indiscrimination between the 12 serotypes, and low sensitivity. In contrast, the VN test represents the gold standard due to its high sensitivity and specificity in differentiating between the 12 FAdV serotypes [73]. However, this assay presents significant trade-offs in terms of cost and time, as well as the necessity of cell culture and reference strains [74]. Because of its technical requirements, the VN test cannot be applied for mass detection.

Advancements in serological techniques have mainly focused on the use of Enzyme-Linked Immunosorbent Assays (ELISA) to detect type- or serotype-specific antibodies of FAdV-1. Initially, the whole CELO virus was used as a coating antigen to establish an ELISA test for FAdV-1 and Adenovirus-Associated Viruses (A-AV) antibody detection [75]. This test has shown a sensitivity level comparable to that of the VN test. Subsequently, significant progress has been made using recombinant proteins for ELISA tests, enabling specific detection of relevant FAdV serotypes including FAdV-1. Different types of recombinant proteins have been used to reach the highest level of specificity and sensitivity. One approach involves the use of both 33 kDa and 100 kDa non-structural proteins of the CELO virus as antigens [76]. The results showed that the 100K-33K-ELISA exhibited a higher sensitivity level than the AGP test and sensitivity and specificity comparable to that of ELISA based on the whole virus. Moreover, the test can be used to distinguish acute FAdV infection from an inactivated virus-based vaccination response, making it suitable for monitoring either disease or vaccination efficacy. Furthermore, two recombinant Fiber-1 and Fiber-2 proteins specific to FAdV-1 and FAdV-4 were separately used as coating antigens to develop an ELISA test that can specifically detect these clinically important serotypes [77]. This test was demonstrated to be specific as no cross-reactions were detected using positive sera of other avian viruses. Additionally, this test displayed equal or even greater sensitivity compared to the VN test while providing advantages in terms of processing and time of analysis, making it suitable for FAdV-1 and FAdV-4 diagnosis in chicken flocks. Another new serological tool is Fluorescent Microsphere Multiplexed Immunoassay (FMIA), which uses recombinant fibers from six distinct FAdV serotypes: FAdV-1, 2, 4, 8a, 8b, and 11 [78]. With specificity comparable to that of the VN test and fiber-based ELISA, the test enabled simultaneous detection of antibodies against all six clinically relevant serotypes in one single reaction with a high-throughput setting.

Early diagnosis remains a critical step in the management of Aviadenovirus infection. Traditional virological techniques including virus isolation have always been considered as the “Gold standard”, even though they are laborious and time-consuming. Other serological tests have the same disadvantages: they are laborious and time-consuming, and some of them are not very sensitive and/or not very specific. As a result, many serological tests are currently being replaced by molecular tests.

### 7.5. Molecular-Based Assay

Today, several molecular techniques have been established for FAdV detection, quantification, and genotyping. These include Restriction Endonucleases Analysis (REA), conventional PCR (cPCR) which is sometimes coupled with Restriction Fragment Length Polymorphism (RFLP), quantitative Real-Time PCR (qPCR), High-Resolution Melting Curve Analysis (HRM), Loop-Mediated Isothermal Amplification (LAMP), Cross-Priming Amplification (CPA), Recombinase Polymerase Amplification (RPA), Dot Blot Assay combined with cPCR, and Amplification Refractory Mutation Systems Quantitative (PCR ARMS-qPCR). Compared to conventional tests, molecular techniques have considerably improved sensitivity, specificity, reproducibility, and analysis time with regard to FAdV diagnosis. These tests can be either universal, enabling the detection of all 12 FAdV serotypes, including FAdV-1, or specific to a single serotype, such as FAdV-1.

Initially, REA was used to cluster and differentiate FAdVs isolates based on the genomic similarities of DNA digested by *Bam*HI and *Hin*dIII restriction enzymes. A total of 12 serotypes from 17 FAdV strains were divided into 5 species (A–E) [5]. Subsequently, cPCR tests were developed based on the use of universal primers targeting conserved regions within the FAdV genome, such as certain regions of the Hexon gene [79], 52K + PIIIa [80], and DNA Polymerase [81]. Most of these universal primers contain degenerate bases including all possible variations between FAdVs serotypes. Primers such as Hexon A/Hexon B, H1/H2, H3/H4, and FAdVF JSN/FAdVR JSN, targeting a conserved region on the Hexon gene, have successfully amplified all FAdV serotypes. However, to identify the serotype, these tests are often combined with RFLP analysis, which uses repeated digestion by restriction enzymes, producing restriction profiles specific to each serotype, including FAdV-1 [79]. Nevertheless, analysis of the HVR1 region revealed numerous differences between strains of the same serotype from different countries and continents, suggesting that the region cannot be used to distinguish strains of the same serotype isolated in different regions [82]. Furthermore, sequencing of the amplified product followed by comparison of the sequence with those available on GeneBank via the Blast bioinformatics tool is often used for FAdV genotyping [82]. Although cPCR followed by sequencing is costly and time-consuming and requires qualified personnel for results analysis, it remains the reference method for FAdV diagnosis and genotyping. To improve the sensitivity and specificity of cPCR tests, a Nested PCR targeting FAdV DNA Polymerase gene has been developed [83]. This technique uses two pairs of primers for two successive PCR reactions. The first primer pair is designed to annex to sequences upstream of the second primer pair in the first PCR reaction. The resulting amplicons are then used as templates for the second primer pair in a second PCR reaction.

On the other hand, specific primers have been meticulously designed to target the Hyper-Variable Region (HVR) of the Hexon gene, enabling specific amplification of FAdV-1 in chicken flocks in Japan [84]. Similarly, a duplex PCR has been developed for the specific detection of FAdV-1 and FAdV-5, where amplified product sizes differentiate between two serotypes in case of co-infection [85]. However, some studies have suggested that PCR-RFLP targeting the long fiber gene could be used to differentiate non-pathogenic from pathogenic strains of FAdV-1 involved in AGE [86]. Despite this, other research has reported similar restriction profiles of the long fiber gene from non-pathogenic (Ote and CELO) and pathogenic strains isolated from chickens with AGE [87].

Alternatively, Real-Time PCR has become a widely used technique in the diagnosis of various pathogens due to its sensitivity, reproducibility, efficiency, and reduced risk of contamination. Unlike conventional PCR, qPCR allows real-time quantification using fluorescent molecules or fluorogenic probes. qPCR assays based on SYBER Green have been developed for FAdV detection and quantification [80]. Universal primers (52K-fw/52K-rv) binding to a conserved region on the 52K gene have proven to be suitable for the accurate detection and quantification of all 12 FAdV serotypes. Additionally, a universal TaqMan probe-based qPCR test has been developed [88]. These qPCR assays have considerably improved sensitivity and analytical precision, reducing the cost, time, and complexity of analysis, as well as the risk of contamination due to post-PCR manipulations. Furthermore, a qPCR test coupled with HRM analysis has been developed. The use of the primer HexL1s/hexL1, targeting a hypervariable region of the Hexon gene, allows universal amplification of the 12 serotypes [89]. Subsequently, HRM analysis of PCR products has been used as a rapid and effective tool for FAdV genotyping, facilitating clinical and epidemiological investigations [90].

With the constant advances in molecular diagnosis, several isothermal techniques, such as LAMP [91], CPA [92], and RPA [88], have been successfully developed for FAdV detection, overcoming many of the limitations of traditional tests. These amplification techniques enable rapid, specific amplification at constant temperatures, eliminating the need for a thermocycler. These characteristics make these techniques ideal for point-of-care applications in resource-limited areas and emergency situations. In addition, a dot blot test based on the PCR method has been developed to overcome the sensitivity limitations of traditional techniques. This test is able to detect all 12 serotypes, with a sensitivity level that is 100 times greater than that of cPCR [93]. Furthermore, ARMS-qPCR has been established for the quantification of and differentiation between the European pathogenic strain (FAdV-1/PA7127) and the apathogenic strain CELO [61]. This method utilizes Single Nucleotide Polymorphisms (SNPs) in the gene encoding for the short fiber protein, enabling accurate discrimination between these strains. These cutting-edge methods have significantly improved the sensitivity, specificity, and analysis time of FAdV detection, providing valuable tools for diagnosis (Table 2).

## 8. Prevention

Adenoviral Gizzard Erosion is considered as an emerging disease that is causing significant losses in the poultry industry. Unfortunately, maternal antibodies do not confer protection against infection with FAdV-1 [43]. Nevertheless, resistance to infection has been observed in chickens after a second infection with the same virulent strain, FAV-99ZH [66]. Protected chickens showed no clinical signs, necrosis, or inclusion bodies. Another study reported that the infection of broilers with the apathogenic strain (CELO) conferred complete protection after provocation with the virulent strain (FAdV-1/PA7127) [61]. However, protected chickens continue to excrete the challenge virus (FAdV-1/PA7127) in their feces. This finding is coherent with the results of a previous investigation reporting excretion of the provocation virus in birds that were clinically fully protected after vaccination with live attenuated FAdV-4 [96]. Similarly, 20-week-old SPF laying hens infected with CELO were protected after being exposed to a virulent strain of FAdV-1 [97]. Although vaccinated chickens were protected, the levels of neutralizing antibodies against FAdV-1 remained low after challenge [66]. These results suggest that, to prevent horizontal infection by FAdV-1, cellular immunity may play a more critical role than humoral immunity. Consequently, it may be concluded that the administration of inactivated and subunit vaccines, which primarily stimulate the production of humoral immune responses, could be an ineffective strategy for preventing FAdV-1 infection. However, the main reason why live vaccines are currently the only candidates against AGE is because they can be orally administered. This administration enables an appropriate local immune response to be triggered directly in the chickens’ gastrointestinal tract, which is crucial for combating the infection.

On the other hand, to reduce economic losses associated with horizontal transmission of FAdV-1, rigorous preventive measures and biosafety protocols, along with surveillance, are recommended. Farmers should implement regular disinfection plans for facilities, equipment, and vehicles, as well as strict control of visitors and personnel to prevent the introduction and spread of the FAdV-1 virus.

## 9. Conclusions

In conclusion, Avian Gizzard Erosion caused by *FAdV-1* is a major concern in many countries, particularly in Asia and Europe. The disease is especially prevalent in broilers, although it is also observed in layers. Unlike other *FAdV* serotypes, the mechanisms underlying *FAdV-1* pathogenesis remain ambiguous. The clinical symptoms of AGE are atypical and manifest as flock disuniformity, weight loss, and sometimes a decrease in egg production, accompanied by mortality. Molecular diagnosis methods, such as conventional PCR followed by restriction enzyme digestion or sequencing, real-time PCR, and PCR followed by melting curve analysis, have greatly improved the diagnosis of *FAdV-1* infections, offering advantages in terms of specificity, rapidity, sensitivity, and efficiency compared to classical techniques like the VN test. Furthermore, transcriptomic profiling of *FAdV-1* infection allows the identification of the genetic and molecular pathways involved in the survival and death of infected chickens, providing new insights into its mechanisms of infection and impact on poultry populations.

## Figures and Tables

**Table 2:** Universal and specific molecular techniques for FAdV-1 diagnosis.

Type	Molecular Assay	Forward Primer	Reverse Primer	Primer Position	Target Gene	Sequence (5′ to 3′)	Product Size (bp)	Test Performance	**Reference**
Universal test	cPCR	52K-F	52K-R	12,788–12,806 13,542–13,526	52K + PIIIa (FAdV-1)	TGT ACG AYT TCG TSC ARA C TARATGGCG CCYTGCTC	755-794	-Universal detection of FAdV.	[80]
	cPCR + RFLP	H1	H2	296–314 1514–1496	Hexon	TGGACATGGGGGGCGACCTA AAGGG ATTGACGTTGTCCA	1291	-Universal amplification of FAdVs. -Differentiation between serotypes using *Hae* II digestion *except* FAdV-5 and FAdV-4.	[94]
	cPCR + RFLP	H3	H4	1501–1522 2819–2801	Hexon (FAdV-1)	AACGT CAACCCCTTCAACCAC CTTGCC TGTGGCGAAAGGCG	1319	-Universal detection of FAdV. -Differentiation between FAdV-1 and other serotypes using *Hpa* II.	[94]
	cPCR + RFLP	HexonA	HexonB	144–161 1041–1021	Hexon (FAdV-1)	CCCTCCCACCGCTTACCA CCCTCCCACCGCTTACCA	900	-Universal detection of FAdV. LOD: 10^2^ copies/mL. -Successive digestion using *Bsi*WI, *Sty*1, and *Mlu*1. *Asp*1, *Bgl*1, and *Sca*1 allow serotype differentiation.	[79]
	cPCR	MK 89	MK 90	811–828 1229–1211	Hexon	CCCTCCCACCGCTTACCA CCCTCCCACCGCTTACCA	418	-Detection of adenovirus from group I, II, III.	[95]
	cPCR + Sequencing	FAdVF JSN	FAdVR JSN	NR	Hexon (FAdV-1)	AATGTCACNACCGARAAGGC CBGCBTRCATGTACTGGTA	830	-Universal cPCR coupled with sequencing for serotype determination. -Used in phylogenetic and geographic analyses of avian adenovirus.	[82]
	Nested PCR	polF outer PolF inner	PolR outer PolR inner	NR	DNA-Polymerase	TNMGNGGNGGNMGNTGYTAYCC GTDGCRAANSHNCCRTABARNGMRTT GTNTWYGAYATHTGYGGHATGTAYGC CCANCCBCDRTTRTGNARNGTRA	321	-Universal cPCR followed by sequencing was initially used to complete panel of DNA Polymerase sequences for FAdV-6, -8b, -7, -8a, -2, -3, -6, -1, and FAdV-11. -Proved to be effective for FAdV detection.	[81]
	Real-Time PCR (Syber Green)	52K-fw	52K-fw	13,075–13,093 13,250–13,232	52K + PIII (FAdV-1)	ATG GCK CAG ATG GCY AAG AGCGCCTGGGTCAAACCGA	176	-Detection and quantification of 12 serotypes. -LOD: 6.73 copies/reaction. -LOQ: 6.73 x 108 copies/reaction. -Efficiency: 98%.	[80]
	PCR/HRM Analysis	Hex L1-s	Hex L1-as	301–323 890–868	Hexon L1	ATGGGAGCSACCTAYTTCGACAT AAATTGTCCCKRAANCCGATGTA	590	-Universal detection of FAdV. -Rapid differentiation between 12 FAdV serotypes using HRM curve analysis.	[89]
	Cross-Priming Amplification (CPA)	FAdV-5a FAdV-2a FAdV-1s	FAdV-4s FAdV-3a	83–106 211–233 130–151 170–192 130–151 193–211	Hexon	ATACTTTGCCATCAAGAATCTGCT AGGTTCACYTGCCGAATAGAC ACGAGTGGGTSCTCAGAAAGGA TCCAGTCTSGGGAACGACCTGC ACGAGTGGGTSCTCAGAAAGGA GATAGAGGCGCCGTCGGCGC	151	-Universal detection of FAdV. -No thermocycler is required. -LOD: 10^−2^ TCID_50_.	[92]
	Recombinase Polymerase Amplification (RPA)	FAdV-RPA Fw	FAdV-RPA Rev	NR	Hexon	CKCCYACTCGCAATGTCACCACCGARAAGGCH TKAHGCTGTASCGCACGCCGRTARCTGTTGGGC	108	- Universal detection of FAdV serotypes. -Rapid detection in 14 min. -No thermocycler is required. -LOD: 0.1 fg.	[88]
Specific test for FAdV-1	cPCR	Flong	Rlong	2812–2831 30,478–30,497	Long fiber (CELO)	TCATGAACGAGGAGGTTG GTTCATTGATGATAC CCC	2382	-Amplification of long fiber of FAdV-1.	[87]
	cPCR	F700	R800	31,201–31,223 31,304–31,323	Short fiber (CELO)	TACGGGCAATTTTGTGAGCTCTA CCCATGGTGGTTGTGTCGAC	1233	-Amplification of short fiber of FAdV-1.	[86,87]
	cPCR	F3	F4	NR	Short fiber (CELO)	GCA TGG CTG ACC AGA AAA TTG TTC AGA CCG TAA CGG	1233	-Amplification of short fiber of FAdV-1.	[86]
	cPCR	F	R	18,691–19,518	Hexon (CELO)	ATTTTCAACACCTGGGTGGAGAGCA CACGTTGCCCTTATCTTGC	828	-Specific amplification of FAdV-1.	[84]
	Duplex PCR	FAdV1 F FAdV5 F	FAdV1R FAdV5 R	NR	Hexon	TTCGAGATCAAGAGGCCAGT GGTCGAAGTTGCGTAGGAAG TACTGCCGTTTCCACATTCA AGCTGATTGCTGGTGTTGTG	227pb for FAdV-1 178 pb for FAdV-5	-Differentiation of FAdV-1 and FAdV-5 in one reaction based on amplification product size. -Highly specific for FAdV-1 and 178pb for FAdV-5. LOD: 0.0001 ng/µL.	[85]
	Real-Time PCR (TaqMan Probe)	FAdV-1F	FAdV-1R	NR	Hexon	TTCGAGATCAAGAGGCCAGT GGTCGAAGTTCGTAGGAAG Probe: AATCCCTACTCCAACACCCC		-Specific amplification of FAAdV-1. -LOD: 8 copies/µL. -R-squared: 0.991. -Efficiency: 95.03%.	[88]
	Amplification Refractory Mutation Systems Quantitative PCR (ARMS-qPCR)	CELO-f PA7127-f	CELO-r PA7127-r	NR	Short fiber	CGTGTTCAATATGAACCAAAACAT C D AGCCGGTGAAGATAGGCCD CGTGTTCAATATGAACCAGAACAC CGCCGGTGAGGATAGGCTD FAM-CCCGAATCGGGAAGCGTAGTAGGG-BHQ1	NR	-Quantification of and differentiation between two FAdV-1 strains (CELO: apathogenic strain and PA7127: European pathogenic strain) by using SNPs in gene coding for short fiber protein.	[61]

K: G/T, Y: C/T, R: A/G, S: C/G, H: A/T/C, N: A/T/C/G, bp: base pair, NR: not reported, FAM: 6-carboxyfluorescein, BHQ-1: Black Hole Quenche, LOD: limit of detection.

## Data Availability

No new data were created or analyzed in this study. Data sharing is not applicable to this article.

## References

[1] Ono M., Okuda Y., Yazawa S., Shibata I., Tanimura N., Kimura K., Haritani M., Mase M., Sato S. (2001). Epizootic Outbreaks of Gizzard Erosion Associated with Adenovirus Infection in Chickens. Avian Dis..

[2] Matczuk A.K., Niczyporuk J.S., Kuczkowski M., Woźniakowski G., Nowak M., Wieliczko A. (2017). Whole genome sequencing of Fowl aviadenovirus A—A causative agent of gizzard erosion and ulceration, in adult laying hens. Infect. Genet. Evol..

[3] King A.M.Q., Adams M.J., Carstens E.B., Lefkowitz E.J. (2012). Family—Adenoviridae. Virus Taxonomy.

[4] Benkő M., Aoki K., Arnberg N., Davison A.J., Echavarría M., Hess M., Jones M.S., Kaján G.L., Kajon A.E., Mittal S.K. (2022). ICTV Virus Taxonomy Profile: Adenoviridae 2022: This article is part of the ICTV Virus Taxonomy Profiles collection. J. Gen. Virol..

[5] Zsák L., Kisary J. (2008). Grouping of Fowl Adenoviruses Based upon the Restriction Patterns of DNA Generated by Bam HI and Hin dIII: Restriction Patterns of DNA Generated by Bam HI and Hind III. Intervirology.

[6] Hess M. (2000). Detection and differentiation of avian adenoviruses: A review. Avian Pathol..

[7] Grafl B., Garcia-Rueda C., Cargill P., Wood A., Schock A., Liebhart D., Schachner A., Hess M. (2018). Fowl aviadenovirus serotype 1 confirmed as the aetiological agent of gizzard erosions in replacement pullets and layer flocks in Great Britain by laboratory and in vivo studies. Avian Pathol..

[8] Lindgren Y., Banihashem F., Berg M., Eriksson H., Zohari S., Jansson D.S. (2022). Gizzard erosions in broiler chickens in Sweden caused by fowl adenovirus serotype 1 (FAdV-1): Investigation of outbreaks, including whole-genome sequencing of an isolate. Avian Pathol..

[9] Dahiya S., Srivastava R.N., Hess M., Gulati B.R. (2002). Fowl Adenovirus Serotype 4 Associated with Outbreaks of Infectious Hydropericardium in Haryana, India. Avian Dis..

[10] Zhang T., Jin Q., Ding P., Wang Y., Chai Y., Li Y., Liu X., Luo J., Zhang G. (2016). Molecular epidemiology of hydropericardium syndrome outbreak-associated serotype 4 fowl adenovirus isolates in central China. Virol. J..

[11] Abghour S., Zro K., Mouahid M., Tahiri F., Tarta M., Berrada J., Kichou F. (2019). Isolation and characterization of fowl aviadenovirus serotype 11 from chickens with inclusion body hepatitis in Morocco. PLoS ONE.

[12] Hosseini H., Langeroudi A.G., FallahMehrabadi M.H., Ziafati Kafi Z., Dizaji R.E., Ghafouri S.A., Hamadan A.M., Aghaiyan L., Hajizamani N. (2020). The fowl adenovirus (Fadv-11) outbreak in Iranian broiler chicken farms: The first full genome characterization and phylogenetic analysis. Comp. Immunol. Microbiol. Infect. Dis..

[13] Laver W.G., Younghusband H.B., Wrigley N.G. (1971). Purification and properties of chick embryo lethal orphan virus (an avian adenovirus). Virology.

[14] Washietl S., Eisenhaber F. (2003). Reannotation of the CELO genome characterizes a set of previously unassigned open reading frames and points to novel modes of host interaction in avian adenoviruses. BMC Bioinform..

[15] San Martín C. (2012). Latest Insights on Adenovirus Structure and Assembly. Viruses.

[16] Chiocca S., Kurzbauer R., Schaffner G., Baker A., Mautner V., Cotten M. (1996). The complete DNA sequence and genomic organization of the avian adenovirus CELO. J. Virol..

[17] Hess M., Cuzange A., Ruigrok R.W., Chroboczek J., Jacrot B. (1995). The avian adenovirus penton: Two fibres and one base. J. Mol. Biol..

[18] Sohaimi N.M., Hair-Bejo M. (2021). A recent perspective on fiber and hexon genes proteins analyses of fowl adenovirus toward virus infectivity—A review. Open Vet. J..

[19] Liu Y., Wan W., Gao D., Li Y., Yang X., Liu H., Yao H., Chen L., Wang C., Zhao J. (2016). Genetic characterization of novel fowl aviadenovirus 4 isolates from outbreaks of hepatitis-hydropericardium syndrome in broiler chickens in China. Emerg. Microbes Infect..

[20] François A., Eterradossi N., Delmas B., Payet V., Langlois P. (2001). Construction of avian adenovirus CELO recombinants in cosmids. J. Virol..

[21] Akello J.O., Kamgang R., Barbani M.T., Suter-Riniker F., Aebi C., Beuret C., Paris D.H., Leib S.L., Ramette A. (2021). Genomic analyses of human adenoviruses unravel novel recombinant genotypes associated with severe infections in pediatric patients. Sci. Rep..

[22] Schachner A., Gonzalez G., Endler L., Ito K., Hess M. (2019). Fowl Adenovirus (FAdV) Recombination with Intertypic Crossovers in Genomes of FAdV-D and FAdV-E, Displaying Hybrid Serological Phenotypes. Viruses.

[23] Thanasut K., Fujino K., Taharaguchi M., Taharaguchi S., Shimokawa F., Murakami M., Takase K. (2017). Genome Sequence of Fowl Aviadenovirus A Strain JM1/1, Which Caused Gizzard Erosions in Japan. Genome Announc..

[24] Domanska-Blicharz K., Tomczyk G., Smietanka K., Kozaczynski W., Minta Z. (2011). Molecular characterization of fowl adenoviruses isolated from chickens with gizzard erosions. Poult. Sci..

[25] Davison A.J., Benkő M., Harrach B. (2003). Genetic content and evolution of adenoviruses. J. Gen. Virol..

[26] Jakab S., Bali K., Homonnay Z., Kaszab E., Ihász K., Fehér E., Mató T., Kiss I., Palya V., Bányai K. (2023). Genomic Epidemiology and Evolution of Fowl Adenovirus 1. Animals.

[27] Aleström P., Stenlund A., Li P., Pettersson U. (1982). A common sequence in the inverted terminal repetitions of human and avian adenoviruses. Gene.

[28] Shinagawa M., Ishiyama T., Padmanabhan R., Fujinaga K., Kamada M., Sato G. (1983). Comparative sequence analysis of the inverted terminal repetition in the genomes of animal and avian adenoviruses. Virology.

[29] Tanimura N., Nakamura K., Imai K., Maeda M., Gobo T., Nitta S., Ishihara T., Amano H. (1993). Necrotizing pancreatitis and gizzard erosion associated with adenovirus infection in chickens. Avian Dis..

[30] Goodwin M.A., Hill D.L., Dekich M.A., Putnam M.R. (1993). Multisystemic adenovirus infection in broiler chicks with hypoglycemia and spiking mortality. Avian Dis..

[31] Abe T., Nakamura K., Tojo T., Yuasa N. (2001). Gizzard erosion in broiler chicks by group I avian adenovirus. Avian Dis..

[32] Okuda Y., Ono M., Shibata I., Sato S. (2004). Pathogenicity of serotype 8 fowl adenovirus isolated from gizzard erosions of slaughtered broiler chickens. J. Vet. Med. Sci..

[33] Lim T.-H., Lee H.-J., Lee D.-H., Lee Y.-N., Park J.-K., Youn H.-N., Kim M.-S., Youn H.-S., Lee J.-B., Park S.-Y. (2011). Identification and virulence characterization of fowl adenoviruses in Korea. Avian Dis..

[34] Li S., Zhao R., Yang Q., Wu M., Ma J., Wei Y., Pang Z., Wu C., Liu Y., Gu Y. (2022). Phylogenetic and pathogenic characterization of current fowl adenoviruses in China. Infect. Genet. Evol..

[35] Mirzazadeh A., Grafl B., Abbasnia M., Emadi-Jamali S., Abdi-Hachesoo B., Schachner A., Hess M. (2021). Reduced Performance Due to Adenoviral Gizzard Erosion in 16-Day-Old Commercial Broiler Chickens in Iran, Confirmed Experimentally. Front. Vet. Sci..

[36] Bulbule N.R., Deshmukh V.V., Raut S.D., Meshram C.D., Mm C. (2016). Pathogenicity and Genotyping of Fowl Adenoviruses Associated with Gizzard Erosion in Commerciallayer Grower Chicken in India. Indian J. Comp. Microbiol. Immunol. Infect. Dis..

[37] Manarolla G., Pisoni G., Moroni P., Daniele G., Sironi G., Rampin T. (2009). Adenoviral gizzard erosions in Italian chicken flocks. Vet. Rec..

[38] Adel A., Mohamed A.A.E., Samir M., Hagag N.M., Erfan A., Said M., Arafa A.E.S., Hassan W.M.M., El Zowalaty M.E., Shahien M.A. (2021). Epidemiological and molecular analysis of circulating fowl adenoviruses and emerging of serotypes 1, 3, and 8b in Egypt. Heliyon.

[39] Kaján G.L., Kecskeméti S., Harrach B., Benkő M. (2013). Molecular typing of fowl adenoviruses, isolated in Hungary recently, reveals high diversity. Vet. Microbiol..

[40] Garmyn A., Bosseler L., Braeckmans D., Van Erum J., Verlinden M. (2018). Adenoviral Gizzard Erosions in Two Belgian Broiler Farms. Avian Dis..

[41] Levkutova M., Levkut M., Herich R., Revajova V., Seman V., Cechova M., Levkut M. (2023). Fowl adenovirus-induced different manifestations of the disease in two consecutive chicken breeding flocks in a poultry hall. Vet. Med..

[42] Ono M., Okuda Y., Shibata I., Sato S., Okad K. (2007). Reproduction of Adenoviral Gizzard Erosion by the Horizontal Transmission of Fowl Adenovirus Serotype 1. J. Vet. Med. Sci..

[43] Grafl B., Aigner F., Liebhart D., Marek A., Prokofieva I., Bachmeier J., Hess M. (2012). Vertical transmission and clinical signs in broiler breeders and broilers experiencing adenoviral gizzard erosion. Avian Pathol..

[44] Yates V.J., Fry D.E. (1957). Observations on a chicken embryolethal orphan (CELO) virus. Am. J. Vet. Res..

[45] Du Bose R.T., Grumbles L.C. (1959). The Relationship between Quail Bronchitis Virus and Chicken Embryo Lethal Orphan Virus. Avian Dis..

[46] Schade B., Schmitt F., Böhm B., Alex M., Fux R., Cattoli G., Terregino C., Monne I., Currie R.J.W., Olias P. (2013). Adenoviral Gizzard Erosion in Broiler Chickens in Germany. Avian Dis..

[47] Domanska-Blicharz K. (2019). Adenoviral Gizzard Erosions in Commercial Layer Chickens. Pak. Vet. J..

[48] Almquist H.J. (1937). Crystals with vitamin K potency. Nature.

[49] Bird H.R., Oleson J.J., Elvehjem C.A., Hart E.B., Halpin J.G. (1937). Relation of Grit to the Development of the Gizzard Lining in Chicks. Poult. Sci..

[50] Bierer B.W., Carll W.T., Eleazer T.H., Barnett B.D. (1966). Gizzard Erosion and Lower Intestinal Congestion and Ulceration Due to Feed and Water Deprivation in Chickens of Various Ages. Poult. Sci..

[51] Harry E.G., Tucker J.F. (1976). The effect of orally administered histamine on the weight gain and development of gizzard lesions in chicks. Vet. Rec..

[52] Daghir N.J., Haddad K.S. (1981). Vitamin B6 in the etiology of gizzard erosion in growing chickens. Poult. Sci..

[53] Sharma R.N., Pandey G.S. (1990). Début d’érosion et d’ulcération du gésier chez des poulets en Zambie. Rev. D’élevage Médecine Vétérinaire Pays Trop..

[54] Okazaki T., Noguchi T., Igarashi K., Sakagami Y., Seto H., Mori K., Naito H., Masumura T., Sugahara M. (1983). Gizzerosine, a New Toxic Substance in Fish Meal, Causes Severe Gizzard Erosion in Chicks. Agric. Biol. Chem..

[55] Hoerr F.J., Carlton W.W., Tuite J., Vesonder R.F., Rohwedder W.K., Szigeti G. (1982). Experimental trichothecene mycotoxicosis produced in broiler chickens by *Fusarium sporotrichiella* var. sporotrichioides. Avian Pathol..

[56] Novoa-Garrido M., Larsen S., Kaldhusdal M. (2006). Association between gizzard lesions and increased caecal Clostridium perfringens counts in broiler chickens. Avian Pathol..

[57] Okuda Y., Ono M., Yazawa S., Shibata I., Sato S. (2001). Experimental infection of specific-pathogen-free chickens with serotype-1 fowl adenovirus isolated from a broiler chicken with gizzard erosions. Avian Dis..

[58] Grafl B., Liebhart D., Günes A., Wernsdorf P., Aigner F., Bachmeier J., Hess M. (2013). Quantity of virulent fowl adenovirus serotype 1 correlates with clinical signs, macroscopical and pathohistological lesions in gizzards following experimental induction of gizzard erosion in broilers. Vet. Res..

[59] Grafl B., Prokofieva I., Wernsdorf P., Dublecz K., Hess M. (2015). Clinical signs and progression of lesions in the gizzard are not influenced by inclusion of ground oats or whole wheat in the diet following experimental infection with pathogenic fowl adenovirus serotype 1. Avian Pathol..

[60] Maletić J., Spalević L., Kureljušić B., Veljović L., Maksimović-Zorić J., Maletić M., Milićević V. (2023). Fowl Adenovirus Infection—Potential Cause of a Suppressed Humoral Immune Response of Broilers to Newcastle Disease Vaccination. Acta Vet..

[61] Grafl B., Prokofieva I., Wernsdorf P., Steinborn R., Hess M. (2014). Infection with an apathogenic fowl adenovirus serotype-1 strain (CELO) prevents adenoviral gizzard erosion in broilers. Vet. Microbiol..

[62] Tan P.K., Michou A.-I., Bergelson J.M., Cotten M. (2001). Defining CAR as a cellular receptor for the avian adenovirus CELO using a genetic analysis of the two viral fibre proteins. J. Gen. Virol..

[63] Taharaguchi S., Kono Y., Ohta H., Takase K. (2007). Putative host cell receptor for fowl adenovirus detected in gizzard. J. Vet. Med. Sci..

[64] Wang W., Liu Q., Li T., Geng T., Chen H., Xie Q., Shao H., Wan Z., Qin A., Ye J. (2020). Fiber-1, Not Fiber-2, Directly Mediates the Infection of the Pathogenic Serotype 4 Fowl Adenovirus via Its Shaft and Knob Domains. J. Virol..

[65] Song Y., Tao M., Liu L., Wang Y., Zhao Z., Huang Z., Gao W., Wei Q., Li X. (2024). Variation in the affinity of three representative avian adenoviruses for the cellular coxsackievirus and adenovirus receptor. Vet. Res..

[66] Ono M., Okuda Y., Shibata I., Sato S., Okada K. (2004). Pathogenicity by Parenteral Injection of Fowl Adenovirus Isolated from Gizzard Erosion and Resistance to Reinfection in Adenoviral Gizzard Erosion in Chickens. Vet. Pathol..

[67] Verma K.C., Malik B.S., Kumar S. (1971). preliminary report on the isolation and characterization of chicken embryo-lethal-orphan (CELO) virus from poultry. Indian J Anim. Sci..

[68] McFerran J.B., Adair B., Connor T.J. (1975). Adenoviral antigens (CELO, QBV, GAL). Am. J. Vet. Res..

[69] Oberoi M.S., Sharma S.N., Maiti N.K. (1990). Detection of avian adenovirus by counterimmunoelectrophoresis. Indian J. Anim. Sci..

[70] Muroga N., Taharaguchi S., Ohta H., Yamazaki K., Takase K. (2006). Pathogenicity of Fowl Adenovirus Isolated from Gizzard Erosions to Immuno-Suppressed Chickens. J. Vet. Med. Sci..

[71] Adair B.M., McFerran J.B., Calvert V.M. (1980). Development of a microtitre fluorescent antibody test for serological detection of adenovirus infection in birds. Avian Pathol..

[72] Saifuddin M., Wilks C.R., Birtles M.J. (1991). Development of an Immunocytochemical Procedure to Detect Adenoviral Antigens in Chicken Tissues. J. Vet. Diagn. Investig..

[73] Grimes T.M., Culver D.H., King D.J. (1977). Virus-Neutralizing Antibody Titers against 8 Avian Adenovirus Serotypes in Breeder Hens in Georgia by a Microneutralization Procedure. Avian Dis..

[74] Thayer S.G. (1998). Serologic Procedures. Isol. Identif. Avian Pathog..

[75] Dawson G.J., Orsi L.N., Yates V.J., Chang P.W., Pronovost A.D. (1980). An Enzyme-Linked Immunosorbent Assay for Detection of Antibodies to Avian Adenovirus and Avian Adenovirus-Associated Virus in Chickens. Avian Dis..

[76] Xie Z., Luo S., Fan Q., Xie L., Liu J., Xie Z., Pang Y., Deng X., Wang X. (2013). Detection of antibodies specific to the non-structural proteins of fowl adenoviruses in infected chickens but not in vaccinated chickens. Avian Pathol..

[77] Feichtner F., Schachner A., Berger E., Hess M. (2018). Development of sensitive indirect enzyme-linked immunosorbent assays for specific detection of antibodies against fowl adenovirus serotypes 1 and 4 in chickens. Avian Pathol..

[78] Feichtner F., Schachner A., Berger E., Hess M. (2018). Fiber-based fluorescent microsphere immunoassay (FMIA) as a novel multiplex serodiagnostic tool for simultaneous detection and differentiation of all clinically relevant fowl adenovirus (FAdV) serotypes. J. Immunol. Methods.

[79] Meulemans G., Boschmans M., Berg T.P., Decaesstecker M. (2001). Polymerase chain reaction combined with restriction enzyme analysis for detection and differentiation of fowl adenoviruses. Avian Pathol. J. WVPA.

[80] Günes A., Marek A., Grafl B., Berger E., Hess M. (2012). Real-time PCR assay for universal detection and quantitation of all five species of fowl adenoviruses (FAdV-A to FAdV-E). J. Virol. Methods.

[81] Wellehan J.F., Johnson A.J., Harrach B., Benkö M., Pessier A.P., Johnson C.M., Garner M.M., Childress A., Jacobson E.R. (2004). Detection and Analysis of Six Lizard Adenoviruses by Consensus Primer PCR Provides Further Evidence of a Reptilian Origin for the Atadenoviruses. J. Virol..

[82] Niczyporuk J.S. (2016). Phylogenetic and geographic analysis of fowl adenovirus field strains isolated from poultry in Poland. Arch. Virol..

[83] Kaján G.L., Sameti S., Benko M. (2011). Partial sequence of the DNA-dependent DNA polymerase gene of fowl adenoviruses: A reference panel for a general diagnostic PCR in poultry. Acta Vet. Hung..

[84] Mase M., Hiramatsu K., Nishijima N., Iseki H., Watanabe S. (2021). Identification of specific serotypes of fowl adenoviruses isolated from diseased chickens by PCR. J. Vet. Med. Sci..

[85] Niczyporuk J., Samorek-Salamonowicz E., Czekaj H. (2010). Incidence and detection of aviadenoviruses of serotypes 1 and 5 in poultry by PCR and duplex PCR. Bull. Vet. Inst. Pulawy.

[86] Okuda Y., Ono M., Shibata I., Sato S., Akashi H. (2006). Comparison of the Polymerase Chain Reaction-Restriction Fragment Length Polymorphism Pattern of the Fiber Gene and Pathogenicity of Serotype-1 Fowl Adenovirus Isolates from Gizzard Erosions and from Feces of Clinically Healthy Chickens in Japan. J. Vet. Diagn. Investig..

[87] Marek A., Schulz E., Hess C., Hess M. (2010). Comparison of the Fibers of Fowl Adenovirus A Serotype 1 Isolates from Chickens with Gizzard Erosions in Europe and Apathogenic Reference Strains. J. Vet. Diagn. Investig..

[88] Zhang J., Liu J., An D., Fan Y., Cheng Z., Tang Y., Diao Y. (2020). A novel recombinase polymerase amplification assay for rapid detection of epidemic fowl adenovirus. Poult. Sci..

[89] Steer P.A., Kirkpatrick N.C., O’Rourke D., Noormohammadi A.H. (2009). Classification of Fowl Adenovirus Serotypes by Use of High-Resolution Melting-Curve Analysis of the Hexon Gene Region. J. Clin. Microbiol..

[90] Steer P., O’Rourke D., Ghorashi S., Noormohammadi A. (2011). Application of high-resolution melting curve analysis for typing of fowl adenoviruses in field cases of inclusion body hepatitis. Aust. Vet. J..

[91] Xie Z., Tang Y., Fan Q., Liu J., Pang Y., Deng X., Xie Z., Peng Y., Xie L., Khan M.I. (2011). Rapid detection of group I avian adenoviruses by a loop-mediated isothermal amplification. Avian Dis..

[92] Niczyporuk J.S., Woźniakowski G., Samorek-Salamonowicz E. (2015). Application of cross-priming amplification (CPA) for detection of fowl adenovirus (FAdV) strains. Arch. Virol..

[93] Hou L., Su Q., Zhang Y., Liu D., Mao Y., Zhao P. (2022). Development of a PCR-based dot blot assay for the detection of fowl adenovirus. Poult. Sci..

[94] Raue R., Hess M. (1998). Hexon based PCRs combined with restriction enzyme analysis for rapid detection and differentiation of fowl adenoviruses and egg drop syndrome virus. J. Virol. Methods.

[95] Xie Z., Fadl A.A., Girshick T., Khan M.I. (1999). Detection of avian adenovirus by polymerase chain reaction. Avian Dis..

[96] Schonewille E., Jaspers R., Paul G., Hess M. (2010). Specific-Pathogen-Free Chickens Vaccinated with a Live FAdV-4 Vaccine Are Fully Protected Against a Severe Challenge Even in the Absence of Neutralizing Antibodies. Avian Dis..

[97] Grafl B., Berger E., Wernsdorf P., Hess M. (2020). Successful reproduction of adenoviral gizzard erosion in 20-week-old SPF layer-type chickens and efficacious prophylaxis due to live vaccination with an apathogenic fowl adenovirus serotype 1 strain (CELO). Vaccine.

